# Asthma Mortality Inequalities in Brazil: Tolerating the Unbearable

**DOI:** 10.1100/2012/625829

**Published:** 2012-05-02

**Authors:** Carolina de Souza-Machado, Adelmir Souza-Machado, Alvaro A. Cruz

**Affiliations:** ^1^Escola de Enfermagem, Universidade Federal da Bahia, Brazil; ^2^ProAR, Núcleo de Excelência em Asma da Universidade Federal da Bahia, Brazil; ^3^Programa de Pós-graduação em Medicina e Saúde, Faculdade de Medicina da Bahia, Universidade Federal da Bahia, Brazil; ^4^Departamento de Biomorfologia, Instituto de Ciências da Saúde, Universidade Federal da Bahia, 40110-100 Salvador, Ba, Brazil

## Abstract

Asthma is responsible for a high morbidity, resulting in hospitalizations, recurrent asphyxiation, and eventually death. In Brazil, where asthma is the third cause of hospitalizations for clinical illnesses and the fourth cause of death from respiratory diseases, some 20% of the population present wheezing. We evaluated the asthma mortality rates in the period between 1998 and 2009, using linear regressions, using the National Mortality Database (Ministry of Health of Brazil). The annual mortality rate (per 100,000 inhabitants) ranged from 1.68 in 1998 to 1.32 in 2009 (mean : 1.49). Brazil presents a slight tendency of reduction in asthma mortality. Asthma mortality rates trends declined in the most developed regions of the country:  Midwest, South, and Southeast, but it increased in the underprivileged regions: North (not statistically significant) and Northeast. This terrible sort of inequality requires urgent reaction from the public health authorities.

Asthma is responsible for a high morbidity, resulting in hospitalizations, recurrent asphyxiation, and eventually death. Worldwide, asthma affects 300 million people most of whom live in low- and middle-income countries. The epidemic of asthma observed in Latin America may continue in the future with increasing urbanization. In Brazil, where asthma is the third cause of hospitalizations for clinical illnesses and the fourth cause of death from respiratory diseases, some 20% of the population present wheezing [1, 2].

We evaluated the asthma mortality rates in the period between 1998 and 2009, using linear regression or general linear regression gamma, using the National Mortality Database (Ministry of Health of Brazil). 31, 843 deaths due to asthma were registered during this period. The annual mortality rate (per 100,000 inhabitants) ranged from 1.68 in 1998 to 1.32 in 2009, mean: 1.49 (Figure 1). Brazil presents a slight tendency of reduction in asthma mortality. Asthma mortality rates trends declined in the most developed regions of the country: Midwest, South, and Southeast, but it increased in the underprivileged regions: North (not statistically significant) and Northeast. Northeast presented the highest increase in asthma deaths during the study period (Table 1). 

Brazil has the 7th greatest economy worldwide [3]. However, less than 7% of GIP have been invested in health resources [4]. This profile added to a poor and unsystematic attention to asthma has resulted in a sharp contrast in the quality of care for this disease in different regions of Brazil. This terrible sort of inequality requires urgent reaction from the public health authorities. Asthma control is possible with simple diagnostic tools and access to recommended inhaled therapy [5]. Health practices for asthma control have been adopted in various countries. The examples of successful programs for asthma control in Finland and Canada demonstrate that it is possible to significantly reduce morbidity, mortality, and costs caused by the disease using simple strategies [5]. 

Since 2002, the Brazilian Ministry of Health have offered free antiasthmatic medications to severe cases; and this policy was extended to others forms of the disease since 2005. However, access to free medication is not enough, unfortunately, because the primary health care professionals are not always prepared to deal with controller therapy of asthma and inhaled medications are irregularly dispensed from public pharmacies. 

What we, lack however, is the recognition of asthma as a major problem. We need to increase awareness of the disease, permanently build capacity of the public health system to improve the diagnosis and to guide regular treatment in primary care, preserving the regional characteristics and striving for simplicity. 

## Figures and Tables

**Figure 1:**
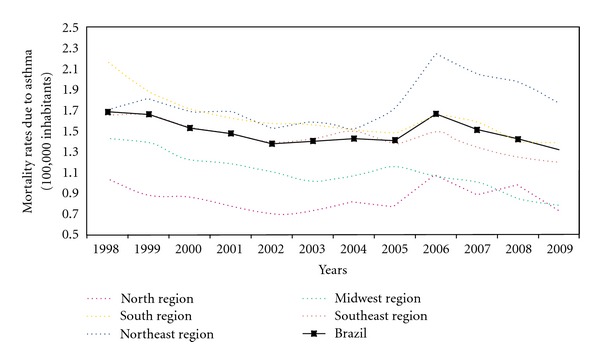
Asthma mortality rates due to asthma, per 100,000 inhabitants, in Brazil and in its regions in the period between 1998 and 2009.

**Table 1:** Mortality rates due to asthma per 100,000 inhabitants in Brazil and in its regions in the period between 1998 and 2009.

Locality	1998	1999	2000	2001	2002	2003	2004	2005	2006	2007	2008	2009	Δ(%)²
Brazil	1.68	1.66	1.53	1.48	1.38	1.40	1.43	1.41	1.67	1.51	1.42	1.32	−21.62
North region	1.04	0.87	0.87	0.78	0.70	0.73	0.82	0.77	1.09	0.89	0.98	0.74	−29.01
Northeast region	1.71	1.81	1.69	1.68	1.53	1.59	1.51	1.70	2.24	2.05	1.98	1.77	3.74
Southeast region	1.65	1.67	1.53	1.47	1.38	1.42	1.52	1.36	1.51	1.34	1.24	1.20	−27.34
South region	2.16	1.87	1.71	1.62	1.57	1.56	1.51	1.48	1.65	1.60	1.40	1.39	−35.90
Midwest region	1.43	1.40	1.22	1.19	1.12	1.01	1.06	1.17	1.06	1.01	0.85	0.78	−45.07

*¹b* value according to linear regression with gamma distribution only for *b* value in Brazil for hole period between 1998 and 2009 (*b* = − 0.016; *P* = 0.0001). ²Percentual difference comparing the first and last years of analyses (1998 and 2009).
